# St. Bartholomew's Hospital

**Published:** 1903-12-05

**Authors:** 


### ST. BARTHOLOMEW'S HOSPITAL.

### THE CONTINUED CONTROVERSY.

**Sir Trevor Lawrence writes, under date November 26th:—**

"With reference to the alleged 'suppressed' report of the Medical Council of this hospital, I shall be much obliged by your allowing me to state that this report was not made to the Governors, and could not, therefore, have been suppressed by them.

"It was prepared for and laid before the Mansion House Committee, and carefully considered by that body, who reported, page 3, paragraph 2, of the enclosed, 'that with the additional land purchased from Christ's Hospital there will be ample room for the provision of a hospital with every modern appliance.'

"I should add that the whole question of the purchase of Christ's Hospital land was thoroughly gone into in 1901 and 1902 by a committee of Governors appointed, in great measure, at the instance of the medical staff, with the result that the Governors decided, on financial grounds, to adhere to the decision already arrived at—that the utmost amount of land they could afford to buy was 1½ acres. The question of the acquisition of more land was again raised on November 5th last by a gentleman who failed to find a seconder in a Court of about 70 Governors.

"The Governors are in constant communication with their staff, to whose advice they naturally attach great value, and I hope to be in a position shortly to ask insertion for a further letter on the subject of our reconstruction plans.

"St. Bartholomew's Hospital, November 26th."

---

**Sir Henry Burdett, replying on November 27th, writes:—**

"The letter of Sir Trevor Lawrence is welcome on public grounds at the moment. It reveals the dangers to the public and the medical profession which underlie the present controversy. It shows that the crisis in the affairs of St. Bartholomew's Hospital has arrived at a point identical with that which occurred at Guy's Hospital a few years ago, when the courage exhibited by the medical staff saved the latter institution from the dangers which threatened it at the time. Briefly put, the matter to be determined in regard to St. Bartholomew's Hospital is whether it shall continue under what I may describe as domestic management without aid from the public, or whether its present form of management shall be changed to one identical with that at Guy's, which gives the medical staff a representative and effective voice in the control of its affairs.

"All true friends of this great hospital must hope that never again in its history will the governors be invited to vote upon a question vital to its efficiency and permanent existence without having submitted to them in their entirety the views of its medical council. The governors have not yet had an opportunity of reading the memoranda giving the full notes of the evidence which the witnesses gave before the Lord Mayor's Committee, and I am in a position to testify that they are still in ignorance of the actual views expressed by some, at any rate, of these expert witnesses. Had the governors had this full evidence and the suppressed report of the medical council before them, it is highly probable that the voting at their meeting on November 5th to which the treasurer refers would have been very different to what it actually was.

The treasurer states that the governors have not suppressed the report of the medical council. This is an amusing way of putting it, seeing that they have had this report withheld from them, and that without it they were not in a position to express an authoritative opinion on the scheme submitted to them. May I inquire who is responsible for the suppression of this report, and of the printed memoranda of the full evidence given before the Lord Mayor's Committee?

"The Mansion House Committee's report quoted by the treasurer, 'that with the additional land purchased from Christ's Hospital there will be ample room for the provision of a hospital with every modern appliance,' is not only in direct conflict with the representations made to that body by the medical council, but it is opposed to the actual possibilities of the site, and can only have been made under grave misapprehensions of what the effect of the present scheme, if carried out, must be upon the hygienic condition of the whole establishment, and upon the permanent reputation and efficient working of the hospital.

"This latter fact is well known to every member of the medical profession who has studied hospital questions, and who understands what a modern hospital must be to-day if it is to fulfil to the fullest extent the requirements of treatment and science. Sir Trevor's letter indicates that the fate of St. Bartholomew's Hospital may, for the moment, rest with the medical council of the hospital. If they adhere to the opinions expressed in the report published in the *Times* of the 26th inst., St. Bartholomew's Hospital will, I am confident, be supplied by the public with adequate funds to increase the present site up to eight and a half acres, the recommendations of the medical council will be carried out, and St. Bartholomew's Hospital will be saved from practical ruin. That these views are held by the medical profession is proved by an article in the *British Medical Journal* of last week, which publishes the architects' plan embodying the scheme of the Lord Mayor's Committee.

"A statement is published to-day to the effect that Mr. Cross, the clerk of the hospital, states that the only thing at present settled is to build an out-patients' department, as shown on the above-mentioned plan. If this be true, it is essential that the medical staff and the public should realise that this department is placed in such a position on the site that, if it is proceeded with, it will render it practically impossible, assuming additional land is purchased up to eight and a half acres, to build new wards under the most favourable conditions the site affords for the accommodation of 700 beds. It would further appear to mean that the architects' plans, as approved by the Lord Mayor's Committee, may be set aside after all by the lay executive of the hospital, in whose hands the decision seems to rest. No rearrangement of the buildings on the present site, involving, as it does, the permanent retention of the old wards and very unsuitable accommodation for the nursing staff, can make the present scheme one which the public can properly be asked to aid by monetary gifts.

"Seeing that in practice the lay executive of the hospital would appear to have dropped plan No. 6 as approved by the Lord Mayor's Committee, might I suggest, in the best interests of the public and the hospital, that they should also withdraw the entire scheme as it stands to-day? They could then set themselves, in conjunction with the medical Council, to unite all parties in formulating a complete plan and scheme for the approval of the citizens at the Mansion House meeting on January 26th, 1904. It is not a question of compromise in any way, so no one's feelings need be ruffled. For there can only be one scheme which, if propounded by a hospital with an assured revenue of something like £100,000 a year, can ever commend itself to the support of the public, and that is to make the only general hospital in the City of London the best modern hospital in the kingdom, and to secure to it a site of adequate proportions to enable our oldest and most famous medical school to fulfil its important mission for the Empire in the completest and fullest possible way.

---

**Sir Trevor Lawrence, under date November 30th, writes:—**

"I regret to find the necessity of writing to you at once forced upon me by Sir Henry Burdett's letter of the 27th inst.

"Your readers can form their own estimate of the statement that the report of the Medical Council was 'suppressed' when I inform them that it was addressed, not to the Governors, but to the Mansion House Committee, and sent, with ten folio pages of other documents, by the Council to everyone of the members—seventeen in number—of that Committee, by whom it was fully considered. But, when the subject of 'suppressions' is raised, it is incumbent upon me to show how little Sir Henry Burdett's statements relating to this hospital are to be trusted. The report in his paper, The Hospital, of the meeting of our Governors on November 5th 'suppressed' the fact that Mr. Motion's amendment, in favour 'of the purchase of more land from Christ's Hospital,' could not even find a seconder in a fully-attended Court of Governors. When Sir Henry Burdett states that the hospital has 'an assured income of something like £100,000 a year,' he 'suppresses' the fact, with which he must have been acquainted, as he has quite recently been furnished with a copy of our last year's accounts, that this is gross income, and is very largely reduced by necessary charges and outlays upon the estates and costs of estate management. The Mansion House Committee, who went exhaustively into the financial affairs of the hospital, reported that the net income of the hospital from all sources for 1902 was £69,586, exclusive of legacies of the exceptional amount of £3,214. They further reported that the effect of purchasing the Christ's Hospital land will begin to be felt to its full extent (£9,033 per annum) in the present year and that a careful forecast showed that, excluding possible legacies, a deficit of from £6,000 to £7,000 a year must be incurred in carrying on the ordinary work of the hospital. Thus Sir Henry Burdett's 'assured income of something like £100,000 a year' dwindles to about £62,000 a year, or by more than one-third.

"What is described as 'domestic management' is management by the Court of Governors, House Committee, and Committee of Treasurer and Almoners, at every one of which representatives of the medical staff have an absolute right to be present. As a matter of fact several members of the medical staff, past and present, attended the Court of Governors on November 5th and expressed their concurrence in the resolutions adopted.

"I do not believe that the Governors of this hospital have any intention of handing over the management of their affairs, which the Mansion House Committee reported unanimously 'had been conducted in a wise and enlightened spirit, with a due regard to economy and in the best interests of the patients,' to Sir Henry Burdett, whose assumption of a sort of papal infallibility would be amusing, were it not injurious to a great charitable institution in need of help from the public.

"Sir Henry Burdett's statement that 'the lay executive would appear to have dropped Plan No. 6, as approved by the Mansion House Committee,' has no foundation. The position of the new out-patient and casualty departments is that recommended by the medical staff, in concert with whom the plans for these departments have been settled.

"The Governors would be only too willing to secure more land, were it not financially impracticable. But the great majority of them are practical business men, and they know that to raise the large sum required for new buildings, etc., will be difficult enough, and that they would be wholly without justification were they to ask the public to give them an additional £320,000 to enable them to buy a further two acres of Christ's Hospital site.

"Such a purchase would bring the cost of land and new buildings together to at least a million.

"Added to this we may reasonably expect an automatic increase to the site available for hospital purposes in the not remote future by the transfer of the teaching of chemistry, physics, and biology, and possibly of anatomy and physiology, to the University of London.

"The spirit in which these attacks on the hospital are being conducted is shown in a letter to the clerk of the hospital, dated November 4th, from Sir Henry Burdett's ally, Mr. Motion, who says, 'We reserve to ourselves the right to oppose to the utmost of our ability the raising of any money to carry it out,' 'it' being the proposals of the Governors and the Mansion House Committee.

"I may add that I have already received donations from several members of the medical staff towards carrying out the present proposals."

---

**Sir Henry Burdett writes, under date December 1st:—**

"Sir Trevor Lawrence's letters show, it seems to me, he entirely fails to apprehend that the discussion we are engaged upon relates to a matter of immense importance to the population of London, and that it cannot be dealt with solely from the point of view of the lay authorities of one hospital, St. Bartholomew's.

"During the last ten years £1,500,000, in round figures, has been spent by certain London hospitals upon new works, renovations, and buildings of various kinds. It is an open secret that within the next ten years St. George's Hospital and King's College Hospital will each require about £300,000, the smaller hospitals some £300,000, and St. Bartholemew's £600,000, to enable the managers to modernise them and make them thoroughly efficient. Then the London hospitals should be able to continue their work with the maximum of efficiency for the next half-century at least.

"Sir Trevor Lawrence and the Mansion House Committee would reduce the expenditure at St. Bartholomew's to £350,000, in defiance of the unanimous testimony of the Medical Council and its recommendations. If Sir Trevor Lawrence was content to leave the Governors to provide this £350,000 out of the resources of St. Bartholomew's Hospital, the public interest in the matter might cease so far as 'Bart's' is concerned. But such a decision would still render it necessary for the public to subscribe the £600,000 that might otherwise go to St. Bartholomew's Hospital, for in such an event it will be imperative to face the facts and shortly to provide London with a new great general hospital, or to add to the resources of one or more of the other general hospitals, so as to supply the void which will be created in our hospital accommodation when the wards of St. Bartholomew's Hospital cease, as they must do within a relatively few years, to be absolutely efficient for the reception of patients.

"My point is that, whereas it is justifiable for St.

Bartholomew's Hospital, which has never before in living memory appealed to the public, to ask for £600,000 to secure an adequate site and to provide our oldest and most renowned medical school with a modern hospital of the most perfect kind, it is not justifiable for the Governors to appeal for funds for a scheme which cannot permanently add to the hospital accommodation of the metropolis. I am well content that my statements should be judged by Sir Trevor's test.

"1. The report of the Governors' meeting in The Hospital of November 14th clearly shows on the face of it that Mr. Andrew Motion's resolution was not seconded.

"2. It is a fact, as stated by me and shown by Sir Trevor Lawrence, 'that St. Bartholomew's Hospital has an assured income of something like £100,000 a year.' The accuracy of this statement is not touched by the useful information supplied by Sir Trevor Lawrence, which shows that of that assured income between 30 and 40 per cent. is expended on 'necessary charges and outlays upon the estates, costs, and estate management.' This is a very hopeful statement from the point of view of the economist and financier, because it raises the hope that it may be possible to reduce these outgoings sufficiently to provide a sum equal to the annual charge which may be necessary to enable St. Bartholomew's Hospital to purchase a further $1\frac{3}{4}$ acres, or thereabouts, to give it an adequate site, and to make it then one of the best general hospitals in the metropolis, by a further expenditure of £350,000. On this head I may state that I should never have ventured to enter upon this discussion had I not felt confident that, whereas it is utterly impossible to expect that the public will subscribe £350,000 to be expended on patchwork and makeshifts, it is reasonably certain that £600,000 may be got to make St. Bartholomew's Hospital worthy of its great traditions and permanently useful as the only general hospital in the City of London.

"The whole basis on which Sir Trevor Lawrence's argument rests is that the Mansion House Committee is 'infallible;' yet that Committee has propounded a scheme in opposition to the unanimous representations of the Medical Council of the hospital, and these representations and the full printed memoranda of the evidence taken by this committee have not yet been submitted to the Governors. On the other hand, I make no 'assumption' of a sort of 'papal' or any other 'infallibility,' but I may be permitted to quote the following paragraph from the conclusions of the Medical Council:—

"'It is generally conceded by all recognised authorities (*vide* Sir Douglas Galton and Sir Henry Burdett) that a *minimum* of 60 patients per acre of site is the standard that should be attained if possible, and the present buildings of St. Thomas's Hospital may be cited as an example of what has been done in recent years in London, where 500 beds with a medical school and nurses' home have been provided for, in about eight acres of ground.'

"Here we get the kernel of the whole matter, and I should welcome the presence of any one who may be interested in the question at the board-room at Charing Cross Hospital, on Thursday next, at 4.30, when I hope to have the pleasure of exhibiting and explaining (1) the plan of the Mansion House Committee which is to cost £350,000, and (2) the plan whereby St. Bartholomew's can be made into a modern hospital of the highest excellency for an outlay, including the extra land, of about £600,000. Such an exposition of facts would seem to indicate that the Mansion House Committee must have been under grave misapprehensions, or they would surely never have propounded plan (1).

"I have little space to deal with the representation of the medical staff in the management, which does not exist in the case of St. Bartholomew's Hospital to-day, as it does at Guy's Hospital. I may point out, however, that the right to be present at meetings without the right to vote has been proved, over and over again, to be useless for any practical purposes in hospital management. Can we have more striking proof of this than the facts (1) that the Mansion House Committee have practically ignored the unanimous representations of the Medical Council of St. Bartholomew's; (2) that the House Committee and the Committee of Treasurer and Almoners have endorsed this action, and (3) that the Court of Governors on November 5th were asked and actually did approve the Mansion House scheme, without first insisting upon the production of the written and matured recommendations of the whole of the Medical Council?

"I am well content to leave the Governors, the medical profession and the public to determine whether those who stand up for the principle that only the best is good enough for our oldest and most renowned medical school and hospital, or those who are content with a scheme which may ultimately ruin both, are entitled to be regarded as the true friends of St. Bartholomew's Hospital."

---

### Mr. Conrad W. Thies writes:—

"I have read with much interest your several articles and Sir Henry Burdett's letters to the *Times* in reference to the proposed improvements at St. Bartholomew's Hospital.

"I entirely agree with the opinion that it would be a shameful waste of charitable funds to expend £350,000 in merely 'tinkering' the present hospital. Many of the existing buildings are structurally defective for the purpose of hospital wards, and no amount spent upon adaptations or additions can bring them up to the standard of sanitary requirements which is essential for a modernly equipped hospital. We have an object lesson in what has been attempted at the London Hospital in this direction during the past few years. As you truly say the present scheme will certainly necessitate further large expenditure in a few years' time.

"Sir Henry Burdett proposes to meet the difficulty by securing more land and erecting entirely new wards and other departments on modern principles at a cost of about £600,000. This proposal is greatly to be preferred to the scheme which has been proposed by the hospital authorities, but I venture to submit the following considerations:—

"In the first place, I think the times are unpropitious for raising such a large sum, unless indeed one of our multimillionaires will immortalise himself by defraying the total cost and thus avoid an appeal to the general public for an honoured institution which has always been regarded as exceptionally well provided for by past endowments.

"The absorption of an exceptionally large sum from charitable funds by any one hospital must always have a prejudicial effect upon the other institutions of the metropolis, because the amount given each year for charitable purposes is fairly constant in amount.

"As you are aware, many of the other hospitals of London are at the present time begging for large sums for new buildings or other improvements, and they are keenly competing with each other in their efforts to raise the funds needed for these purposes. The total amount thus being asked for cannot be less than £1,000,000.

"In view of the existing conditions it is surely time to pause and consider the problem of medical relief and education from a broader standpoint. The other nations of Europe have already realised that the question must be dealt with as a whole and they have recognised the desirability of treating the sick as far as practicable in the purer air of the country rather than in the exhausted air of their cities.

"As was recently stated in The Hospital the city of Paris has decided to pull down the "Hotel Dieu" and the other old Hospitals in the city and to rebuild them on modern principles in the suburbs where space can be obtained at moderate cost.

"On the other hand, we in London continue to expend each year large sums in enlarging and adding to our present hospitals. King's College Hospital is setting a good example in deciding to remove to the suburbs, and I feel confident that in the course of a very few years both the medical profession and public opinion generally will condemn our present arrangements.

"If, therefore, instead of expending such a large sum upon extending their present site, the authorities of St. Bartholomew's had decided to spend a smaller amount in erecting an entirely new hospital somewhere on the outskirts of London, they might have devoted their present site to a new out-patient department with sufficient modernly-constructed wards for all emergency or trivial cases. By some such arrangement they would have been able to provide for a much larger number of patients at a comparatively small outlay.

"I fully realise that any such proposal raises several difficult problems in reference to the medical staff and students, but in these days of rapid locomotion I do not believe that the difficulties are insuperable, while it is certain that patients, students, and nurses would all benefit greatly in health by the change.

"Anyone who has visited Christ's Hospital since its removal from Newgate Street to Horsham will not need any further demonstration of the benefits derivable from fresh air than is evident from the impoved appearance of the Blue Coat boys."

### ST. BARTHOLOMEW'S HOSPITAL NOTICES.

We are requested to state that a copy of the suppressed report of the Medical Council to the Lord Mayor's Committee, together with the architect's plan for reconstruction on the present site, may be obtained free on application or by post on enclosing two stamps to the Manager, The Scientific Press, 28 and 29 Southampton Street, Strand, W.C.

A donation of £2,000 has been received from the Grocers' Company for the out-patients' department of St. Bartholomew's Hospital.

A donation of £1,000 has been received from the Worshipful Company of Mercers to the Building Fund of St. Bartholomew's Hospital.

